# Supplementary material for: MetaRibo-Seq measures translation in microbiomes
Source: Nat Commun. 2020 Jun 29;11:3268. doi: 10.1038/s41467-020-17081-z (PMC7324362; doi:10.1038/s41467-020-17081-z)
Supplement: Supplementary file 10 — Supplementary Data 7 [file 41467_2020_17081_MOESM10_ESM.zip › File2/Confidence_High_Taxonomy/168837_out.krona.html]

Javascript must be enabled to view this page.

members
magnitude
magnitudeUnassigned
count
unassigned
taxon
rank

168837\_out

2

2
2
superkingdom

phylum
2
1239

class
2
186801

186802
2
order

family
31979
2

genus
1485
2

2293021

SRS077641\_contig\_number\_14655
1
species

2293012

SRS142599\_contig\_number\_contig-100\_27956.74583
1
species
